# Retrospective analysis of the immunogenic effects of intra-arterial locoregional therapies in hepatocellular carcinoma: a rationale for combining selective internal radiation therapy (SIRT) and immunotherapy

**DOI:** 10.1186/s12885-020-6613-1

**Published:** 2020-02-19

**Authors:** Ligia Craciun, Roland de Wind, Pieter Demetter, Valerio Lucidi, Ali Bohlok, Sébastien Michiels, Fikri Bouazza, Michael Vouche, Ilario Tancredi, Gontran Verset, Soizic Garaud, Céline Naveaux, Maria Gomez Galdon, Karen Willard Gallo, Alain Hendlisz, Ivan Duran Derijckere, Patrick Flamen, Denis Larsimont, Vincent Donckier

**Affiliations:** 1 Pathology, Institut Jules Bordet, Université Libre de Bruxelles, Bruxelles, Belgium; 2 Pathology, Hôpital Erasme, Université Libre de Bruxelles, Bruxelles, Belgium; 3 Abdominal Surgery, Hôpital Erasme, Université Libre de Bruxelles, Bruxelles, Belgium; 4 Surgery, Institut Jules Bordet, Université Libre de Bruxelles, Rue Héger-Bordet, 1, B-1000 Brussels, Belgium; 5 Radiology, Institut Jules Bordet, Université Libre de Bruxelles, Bruxelles, Belgium; 6 Radiology, Hôpital Erasme, Université Libre de Bruxelles, Bruxelles, Belgium; 7 Gastroenterology and Medical Oncology, Hôpital Erasme, Université Libre de Bruxelles, Bruxelles, Belgium; 8 Molecular Immunology Unit, Institut Jules Bordet, Université Libre de Bruxelles, Bruxelles, Belgium; 9 Medical Oncology, Institut Jules Bordet, Université Libre de Bruxelles, Bruxelles, Belgium; 10 Nuclear Medicine, Institut Jules Bordet, Université Libre de Bruxelles, Bruxelles, Belgium

**Keywords:** Hepatocellular carcinoma, Selective internal radiation therapy (SIRT), Transarterial chemoembolization (TACE), Immunotherapy, Tumor-infiltrating lymphocytes, Locoregional therapy

## Abstract

**Background:**

Immunotherapy represents a promising option for treatment of hepatocellular carcinoma (HCC) in cirrhotic patients but its efficacy is currently inconsistent and unpredictable. Locoregional therapies inducing immunogenic cell death, such as transarterial chemoembolization (TACE) or selective internal radiation therapy (SIRT), have the potential to act synergistically with immunotherapy. For the development of new approaches combining locoregional treatments with immunotherapy, a better understanding of the respective effects of TACE and SIRT on recruitment and activation of immune cells in HCC is needed. To address this question, we compared intra-tumor immune infiltrates in resected HCC after preoperative treatment with TACE or SIRT.

**Methods:**

Data fromr patients undergoing partial hepatectomy for HCC, without preoperative treatment (SURG, *n* = 32), after preoperative TACE (TACE, *n* = 16), or preoperative SIRT (*n* = 12) were analyzed. Clinicopathological factors, tumor-infiltrating lymphocytes (TILs), CD4^+^ and CD8^+^ T cells, and granzyme B (GZB) expression in resected HCC, and postoperative overall and progression-free survival were compared between the three groups.

**Results:**

Clinicopathological and surgical characteristics were similar in the three groups. A significant increase in TILs, CD4^+^ and CD8^+^ T cells, and GZB expression was observed in resected HCC in SIRT as compared to TACE and SURG groups. No difference in immune infiltrates was observed between TACE and SURG patients. Within the SIRT group, the dose of irradiation affected the type of immune infiltrate. A significantly higher ratio of CD3^+^ cells was observed in the peri-tumoral area in patients receiving < 100 Gy, whereas a higher ratio of intra-tumoral CD4^+^ cells was observed in patients receiving > 100 Gy. Postoperative outcomes were similar in all groups. Irrespective of the preoperative treatment, the type and extent of immune infiltrates did not influence postoperative survival.

**Conclusions:**

SIRT significantly promotes recruitment/activation of intra-tumor effector-type immune cells compared to TACE or no preoperative treatment. These results suggest that SIRT is a better candidate than TACE to be combined with immunotherapy for treatment of HCC. Evaluation of the optimal doses for SIRT for producing an immunogenic effect and the type of immunotherapy to be used require further evaluation in prospective studies.

## Background

Either for curative-intent or for palliative approaches, therapeutic management of hepatocellular carcinoma (HCC) in cirrhotic patients remains limited by the poor efficacy of currently available systemic therapies. In particular, potentially curative treatments, such as partial hepatectomy (PH) and destruction with radiofrequency (RF), are associated with high recurrence rates [1–4], strongly underlining the rationale for combining tumor-targeted approaches with effective systemic therapy. At the present, however, no neoadjuvant or adjuvant systemic treatment has been proven effective for reducing the risk of relapse when combined with PH or RF [5–7]. In this context, the use of immunotherapy appears to be an attractive option. Several studies have indicated that HCCs are immunogenic and immunosensitive tumors and promising results have been obtained with different types of immunotherapy, including adoptive immunotherapy and checkpoint inhibitors [8–18]. However, in individual patients, the overall results of immunotherapy remain inconsistent and outcomes are difficult to predict. To address this problem, it has been hypothesized that the reliability and robustness of response to different immunostimulating approaches could be enhanced by combination with loco-regional intra-arterial treatment, such as transarterial chemoembolization (TACE) or selective internal radiation therapy (SIRT). In fact, it has been shown that in situ cellular destruction, such as that induced by TACE, SIRT, or by thermic destruction with RF, may enhance tumor immunogenicity (so-called immunogenic cell death) by increasing the expression of tumor-associated antigens and the recruitment and diversity of tumor-infiltrating lymphocytes (TILs) [19–25]. In that sense, TACE and SIRT may potentially act synergistically with immunotherapy, both locally and for inducing a systemic anti-tumor immune response.

At this stage, both transarterial chemoembolization (TACE) and selective internal radiation therapy (SIRT) are potential candidates for use in such combined approaches. In current algorithms, TACE and SIRT are now similarly validated in the multimodal treatment of HCC, mainly as bridge therapy before transplantation or for palliation in patients not amenable to curative therapies [1]. However, the mechanisms of action of these two techniques are different, as TACE consists of intra-tumor deposition of particles loaded with chemotherapy combined with embolization of small tumor arteries, essentially leading to ischemic cell death, while SIRT consists of intra-tumor deposition of particles loaded with ^90^Yttrium (^90^Y), via smaller arterial branches, leading to radiation-induced cell death. Accordingly, a better understanding of the respective effects of TACE and SIRT on tumor immunogenicity and the tumor immune microenvironment represents a necessary first step for the design of new therapeutic protocols combining intra-arterial therapy and immunotherapy.

To evaluate this question, we analyzed tumor samples from patients who underwent PH for HCC after preoperative TACE or preoperative SIRT, and in patients who underwent surgery without preoperative treatment as a control. In these three groups, we compared immune infiltrates, such as TIL phenotypes and extent, and the intra-tumor expression of the cytotoxic molecule granzyme B (GZB).

## Methods

### Patients

We retrospectively analyzed three groups of patients who underwent PH for HCC, including patients who underwent surgery without preoperative treatment (SURG), and patients who underwent surgery after preoperative TACE (TACE) or after preoperative SIRT (SIRT). All patients had been previously declined for liver transplantation by multidisciplinary board decision. Selection criteria for PH included compensated Child-Pugh A cirrhosis, the absence of significant portal hypertension as defined by a portosystemic gradient ≤10 mmHg, the absence of extrahepatic tumors, resectable disease as defined by the surgical team, and the absence of any contraindication for general anesthesia and liver surgery.

### Preoperative treatments

The decision to use preoperative treatment or not was determined according to the clinical protocols at the time of the patient’s treatment (period: 2012–2017). Patients treated with preoperative SIRT were included in a prospective trial to evaluate the feasibility and safety of SIRT before surgery for HCC in cirrhotic patients (ClinicalTrials.gov NCT01686880) [26]. Two modalities were used for TACE during the period of this study. In conventional TACE, an emulsion of doxorubicin (1 mg/kg) in lipiodol (iodinated poppy seed oil, Guerbet, France) was injected intra-arterially, followed by gelfoam embolization. For drug-eluting bead TACE, Dc Bead™ 100–300 μm (Biocompatibles UK Ltd) or LifePearl™ 200 μm (Terumo Europe NV, Leuven, Belgium) beads loaded with 75 mg doxorubicin were injected intra-arterially and followed by embolization with an embolic agent (gelfoam particles or Bead Block™, Biocompatibles, UK Ltd).

SIRT was performed as a 2-step procedure. First, a simulation was performed to assess the feasibility and safety of the treatment using the injection of 99mTc-labeled macro-aggregated albumin via supra-selective catheterization of the tumor-feeding segmental or lobar artery in a position determined according to pre-simulation contrast-enhanced angio-CT scan. SIRT was performed when the simulation showed tumor targeting and in the absence of significant pulmonary or gastrointestinal dissemination (pulmonary shunting ≤10%, according to safety requirements outlined by Lemaire et al.) [26]. In the second step, in the absence of contraindication, intra-arterial ^90^Yttrium (^90^Y) resin microspheres SIR-Spheres (Sirtex Medical Limited, Sidney, Australia) were injected supra-selectively into the position defined during SIRT simulation.

### Surgery

In order to obtain a substantial biological effect of preoperative treatment and for radioprotection when the patients received SIRT, PH was planned at least 12 weeks after TACE or SIRT. PH was performed via laparoscopic or open approach according to technical considerations and surgeon’s choice. All resections were performed under intraoperative ultrasound guidance with the aim of achieving a tumor-free margin. Resections of three or more liver segments were defined as major hepatectomies. Postoperative complications were graded according to Clavien classification [27].

### Pathology and evaluation of pathological response

HCCs were graded as well, moderately, or poorly differentiated. To evaluate the response to preoperative treatments, tumor necrosis was analyzed using hematoxylin eosin slides. Minor, moderate, and major pathological responses were defined when tumor necrosis was < 25%, ranging from 25 to 50%, and > 50%, respectively. No response and complete response were defined by the absence of necrosis and the absence of residual cancer cells, respectively.

### Immunohistochemistry staining

Consecutive formalin-fixed paraffin-embedded tissue sections (4 μm) were immunohistochemically (IHC)-stained using a BenchMark XT IHC/ISH automated slide stainer (Ventana Mediated Systems) using the Ventana Benchmark technology-based detection system (Roche). Dual CD3/CD20, IHC stains were performed as previously described [28], using primary antibodies against CD3 (Abcam) and CD20 (DAKO). CD4, CD8, and GZB IHC were performed as single stains using monoclonal antibodies against CD4 (BioSB), CD8 (DAKO), and GZB (Abcam).

### Image acquisition

Digital images were captured using the NanoZoomer 2.0- RS slide scanner (Hamamatsu, Japan) under 40x magnification.

### Digital image analysis

Immunohistochemically stained sections were scanned at a magnification of 20x on a NanoZoomer slide scanner (Hamamatsu). Images were analyzed using Visiomorph^DP^ software (Visiopharm) to quantify the CD3^+^, CD4^+^, CD8^+^ T cells, and GZB signals within the tumor and stromal areas defined by a pathologist for each digital image. The necrotic areas were manually excluded. All images were visually reviewed to remove staining artefacts and damaged tissue area. The total positively stained area(s) was scored as a percentage of the defined region.

### Survival and correlation analyses

To investigate the potential effects of the type and extent of TILs and intratumoral expression of GZB, individual CD3+, CD4+, CD8+, and GZB data were correlated with disease-free survival (DFS), as defined by the time from surgery until the first documented progression of disease, and with overall survival (OS).

### Lesion dosimetry

In patients treated with SIRT, lesion dosimetry was performed either on pre-therapeutic ^99^Tc-MAA SPECT/CT images or post-therapeutic ^90^Y-PET/CT using PMOD® (Technologies Ltd.; Zurich, Switzerland). Lesions were manually delineated on the anatomical images by an experienced physician and co-registered with either pre-therapeutic or post-therapeutic images.

### Statistics

The data were analyzed using the SPSSv25 software. Clinicopathologic parameters were compared between SURG, TACE, and SIRT groups, using the ANOVA test for continuous data and the Chi-Square test for categorical variables. OS and DFS were defined as the time from the date of surgery to the date of death, from any cause, and to the date of the detection of recurrence or death, whichever occurred first, respectively. Survival curves were generated using the Kaplan-Meier method and the comparison between the three groups was performed using the Log-Rank test. The correlations between the extent of TIL and GZB intratumor expression and OS or PFS were evaluated using Cox regression analysis. In all tests, a *p* < 0.05 defined statistical significance.

## Results

### Patient and surgical characteristics and post-surgical outcomes

Overall, patient and tumor characteristics were similar in the three groups (Table 1). No differences were observed in demographics and causes/stages of cirrhosis between the SURG (*n* = 32), TACE (*n* = 16), and SIRT (*n* = 12) groups (Table 1). Patients were predominantly male, the median age of the entire population was 67.7 years (range: 27–88 years), and most of the patients had hepatitis C and alcohol-related cirrhosis. All patients were Child-Pugh A. The median tumor number was 1 in the three groups, and median tumor size was similar in the three groups, 40, 49.5, and 47.5 mm in SURG, TACE, and SIRT, respectively. No patients had macrovascular tumor invasion at imaging. Alfa-fetoprotein (AFP) levels varied widely but were not significantly different among the three groups. The median delta AFP level, as measured between dosage at diagnosis and preoperative dosage after TACE or SIRT, was similar in the TACE and SIRT groups (data not shown). The time interval between TACE or SIRT and surgery was similar, 14.4 and 15 weeks, respectively. Surgical approaches were similar in the three groups, consisting of laparoscopic resection in 34, 37, and 33% in SURG, TACE, and SIRT groups, respectively (Table 1). Significantly more patients underwent a major hepatectomy in the TACE group, representing 69%, as compared with 15 and 25% in the SURG and SIRT groups, respectively. One postoperative death was observed in the SURG group, 1 in the TACE group, and 0 in the SIRT group. Operative complication rates were similar in the three groups (Table 1).

**Table 1:** Patient, cirrhosis, tumor, and surgical characteristics

	Surgery (*n* = 32)	TACE (*n* = 16)	SIRT (*n* = 12)	*P*
Male sex, n (%)	19 (65.5%)	14 (87.5%)	11 (91.5%)	0.075
Mean age in years (median, range)	69.5 (65.5, 27–83)	67 (65.1, 29–88)	64 (62.6, 44–73)	0.8
Cause of cirrhosis^a^, *n* (%)				
Alcohol	11 (34.5%)	7 (43.75%)	3 (25%)	0.75
Virus	13 (40.5%)	9 (56.25%)	8 (66.5%)	0.5
HCV	8 (25%)	7 (43.75%)	7 (58.5%)	
HBV	3 (9.5%)	2 (12.5%)	1 (8.5%)	
HBV + HCV	2 (6.25%)	0	0	
Other	4 (12.5%)	2 (16.5%)	1 (8.5%)	
Mean MELD (median, range)	8.7 (7.9, 6.4–18.6)	9.1 (9.1, 6.4–13.8)	6 (7.7, 6–9.1)	0.2
Mean tumor number (median, range)	1.1 (1, 1–3)	1.3 (1, 1–3)	1.7 (1, 1–2)	0.4
Mean tumor diameter (mm) (median, range) mm	53.5 (40, 13–150)	59.6 (49.5, 30–150)	61.6 (47.5, 18–150)	0.75
Mean AFP level (ng/ml) (median, range)	571 (19.7, 2–9900)	4267 [195, 1–69,000)	10,883 (38.5, 3–121,000)	0.281
AFP > 400 ng/ml	3 (9.5%)	5 (31.25%)	3 (25%)	0.36
Laparoscopic resection	11 (34.5%)	6 (37.5%)	4 (33.5%)	0.96
Major resection	5 (15.5%)	11 (68.75%)	3 (25%)	0.006
Operative mortality	1 (3.1%)	1 (6.25)	0	
Operative morbidity	13 (40.5%)	7 (43.75%)	5 (41.5%)	0.5
0	19 (59.5%)	9 (56.25%)	7 (58.3%)	
1	4 (12.5%)	0	1 (8.5%)	
2	5 (15.5%)	5 (31.25%)	3 (25%)	
3a	2 (6.25%)	1 (6.25%)	1 (8.5%)	
3b	2 (6.25%)	1 (6.25%)	0	
4	0	0	0	

^a^Some patients could have several causes of cirrhosis

*TACE* transarterial chemoembolization, *SIRT* selective internal radiation therapy, *HBV* hepatitis B virus, *HCV* hepatitis C virus, *MELD* model for end-stage liver disease, *AFP* alpha-fetoprotein

### Pathological data

The tumor histologic grades were similar in the three groups (Table 2). Significantly increased rates of tumor necrosis were observed in the TACE and SIRT groups as compared with spontaneous necrosis in the SURG group, including 28 and 17% complete responses, respectively (Table 2).

**Table 2:** Pathological data

	SURG (*n* = 32)	TACE (*N* = 16)	SIRT (*N* = 12)	*P*
Tumor differentiation				0.51
Well	10 (31.2%)	5 (31.2%)	3 (25%)	
Moderate	18 (56.2%)	10 (62.5%)	6 (50%)	
Poorly	4 (12.5%)	1 (6.25%)	3 (25%)	
Tumor necrosis				< 0.001
Absence	26 (81.2%)	1 (6.25%)	3 (25%)	
Minor (1–25%)	4 (12.5%)	2 (12.5%)	1 (8.3%)	
Moderate (25–50%)	2 (6.25%)	3 (18.75%)	2 (16.6%)	
Major (> 50%)	0	5 (31.2%)	4 (33.3%)	
Complete (100%)	0	5 (31.2%)	2 (16.6%)	

*SURG* surgery, *TACE* transarterial chemoembolization, *SIRT* selective internal radiation therapy

### Intra-tumor infiltrating lymphocytes and granzyme B expression

**Fig. 1:**
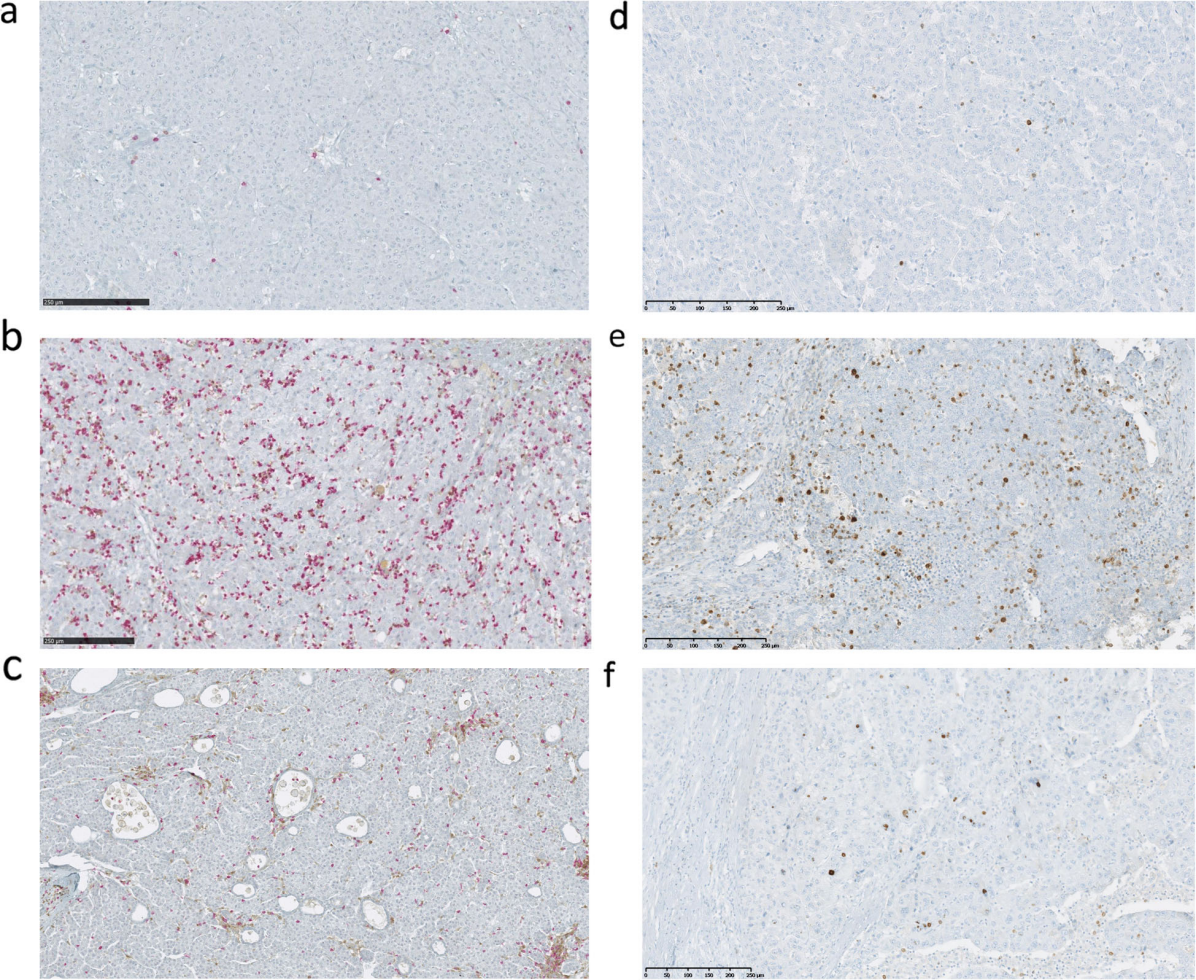
Representative images of dual CD4/ CD8 and Granzyme B staining on tumor tissues. Scans were imaged at 10x magnification using NDPview software (Hamamatsu). **a** TILs in a non-treated HCC patient, showing CD4^+^ (brown) and CD8^+^ (red) cells. **b** TILs in a preoperative SIRT-treated HCC patient, showing increased infiltrates with CD4^+^ (brown) and CD8^+^ (red) cells. **c** TILs expression in a preoperative TACE-treated HCC patient showing similar infiltrates with CD4^+^ (brown) and CD8^+^ (red) cells as observed in untreated patients but associated with significant areas of necrosis. **d** Granzyme B expression in a non-treated HCC patient (brown). **e** Granzyme B expression in a preoperative SIRT-treated HCC patient. **f** Granzyme B expression in a preoperative TACE-treated HCC patient

**Fig. 2:**
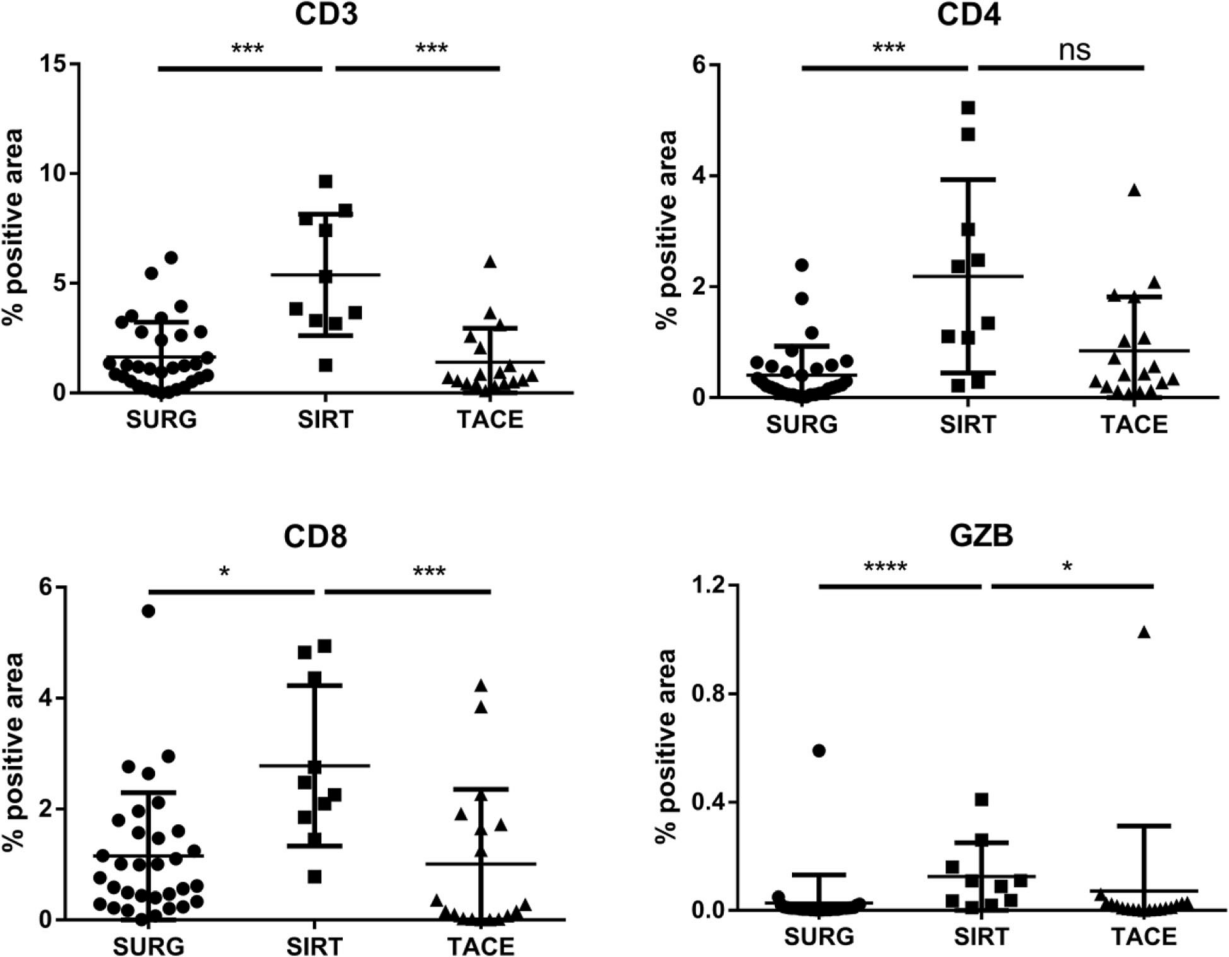
Increased TIL and Granzyme B expression in patients treated preoperatively with SIRT as compared with patients treated preoperatively with TACE and patients receiving no preoperative treatment. Comparison of CD3+, CD4+, CD8+, and Granzyme B in the three groups of patients. Each dot represents one individual. *: *p* < 0.05, ***: *p* < 0.01

Digital pathology was used to quantify TILs and granzyme B expression on scanned CD3, CD4/CD8, and GZB IHC-stained tissues. The resulting data demonstrated significant modifications of the immune infiltrates in SIRT patients as compared with TACE and SURG (Figs. 1 and 2). A significant increase in CD3^+^ TILs was observed in SIRT patients as compared with TACE and SURG patients, including a significantly increased ratio of both CD4^+^ T helper cells and CD8^+^ cytotoxic cells (Fig. 2). In contrast, preoperative TACE did not significantly modify TIL numbers and subsets as compared with the untreated condition in SURG patients (Fig. 2). Moreover, significant intra-tumoral expression of GZB was observed in SIRT as compared with SURG and TACE patients, while no modification was demonstrated between TACE and SURG groups (Fig. 2). Among SIRT patients, we compared TILs and GZB expression between patients receiving irradiation < 100 Gy (*N* = 6) and those receiving irradiation > 100 Gy (*N* = 6). A significantly higher ratio of CD3^+^ cells was observed in the peri-tumoral area in patients treated with lower doses (*p* = 0.004), whereas a higher ratio of intra-tumoral CD4^+^ cells was observed in patients treated with higher doses (*p* = 0.030) (data not shown). The other T cell populations and GZB expression were not significantly modulated according to different absorbed doses.

### Correlations between intra-tumoral infiltrates and survival

After a mean follow-up of 47, 42, and 35 months, no differences were observed in OS and DFS in the SURG, TACE, and SIRT groups, respectively. In the SURG, TACE, and SIRT groups, mean OS was 72 months (range: 54–93), 73.9 months (range: 50–98), and 74 months (range: 50–98), and mean DFS was 29 months (15–42), 40.5 months (range: 22–55), and 26.3 months (range: 7–47), respectively. To further analyze the potential effect of TILs and intra-tumoral expression of GZB, the percentages of these infiltrates, independently of preoperative treatment, were analyzed according to a Cox regression model. In these analyses, the percentages of CD3^+^ TILs (HR: 1.13, *p* = 0.32), CD4^+^ TILs (HR: 1.04, *p* = 0.15), CD8^+^ TILs (HR: 1.15, *p* = 0.15), and GZB (HR: 1.02, *p* = 0.84) were not correlated with DFS.

## Discussion

There is a strong need to develop new therapeutic strategies for hepatocellular carcinoma in cirrhotic patients. In fact, in most cases, due to the frequency of occult intrahepatic metastases and to the pre-neoplastic nature of the whole cirrhotic liver, HCC should be considered to be a multicentric tumor. Therefore, besides liver transplantation, tumor-targeted approaches, such as PH and RF, and locoregional treatments, such as TACE, SIRT, or radiotherapy, are associated with high recurrence rates and poor long-term results. The combination of immunotherapy with local treatments may represent a new option for addressing this problem [29–31]. Different combinations are possible in this setting, both for the choice of the locoregional treatment and for the type of immunotherapy, and several of these strategies are currently being investigated [32–37]. Among locoregional treatments, both TACE and SIRT have the potential to induce local immunogenic cell death and to improve the efficacy of immunotherapy by revealing new tumor antigenic targets, creating acute inflammatory changes in the tumor microenvironment, and promoting the activation/recruitment of immune-active cells. In curative-intent approaches, it is also possible that the combination of preoperative intra-arterial treatments with immunotherapy may improve the long-term results of PH or RF by inducing a vaccinal-type effect and enhancing immune surveillance to prevent the development of micrometastases or of de novo HCC in the remnant cirrhotic liver.

The central objective of our study was to characterize the immunogenic effects of TACE and SIRT. To evaluate this question, we reviewed tumor samples in cirrhotic patients who underwent PH for HCC after preoperative TACE, preoperative SIRT, and without preoperative treatment. Our main finding is that SIRT, but not TACE, significantly enhances intra-tumor immune infiltrates in HCC as compared with the spontaneous infiltrates observed in patients who were untreated before tumor resection. In SIRT patients, we observed a significant increase in CD3^+^TILs, particularly of CD8^+^T cell subsets. Similarly, but to a lesser extent, a significant increase in CD4^+^TILs was observed in these cases but, as the phenotype of these cells was not characterized (including the relative proportions of regulatory FoP3^+^ and cytotoxic GZB^+^ among CD4^+^T cells), the impact of this phenomenon is difficult to interpret. Consistent with this finding, as CD8^+^T cells represent its major cellular source, a significant increase of GZB was observed in the SIRT group. Overall, these results confirm the recent observation from Chew et al. that showed that radioembolization increases anti-tumor immune responses in HCC [38]. Similarly, these authors showed that SIRT significantly increases CD8^+^T cell intra-tumoral infiltrates, but also other cellular populations involved in anti-tumor immunity, such as NK cells.

The mechanisms leading to immune activation after SIRT remain hypothetical. In fact, several observations have suggested that some of the clinical effects of radiation therapy could be related to stimulation of the antitumor immune response, including in the rare cases where a response is induced at distant sites after radiotherapy (the so-called abscopal effect) [39–41]. More specifically, it has been shown that tumor irradiation may increase the release of tumor antigens and the diversity and activity of TILs [42], suggesting potential synergistic activity with immunotherapy, such as immune checkpoint inhibitors [32].

Interestingly, we also observed that different doses of irradiation delivered during SIRT may differentially affect the type and extent of immune infiltrates and, in particular, that lower doses of irradiation could be more immunogenic. These preliminary observations should be further confirmed but are consistent with results obtained with external beam radiotherapy showing that immune responses are highly dependent on the administered radiation dose and fractioning [43].

In contrast to patients receiving preoperative SIRT, and despite significant amounts of tumor necrosis, no change in immune infiltrates was observed in patients treated with TACE. CD8^+^ and CD4^+^ TILs and GZB intra-tumor expression were not different in this group as compared with the spontaneous condition in untreated tumors, indicating that, in these conditions, TACE-related ischemic cell death does not generate significant modification of the inflammatory/immunogenic tumor microenvironment. This discrepancy between cellular necrosis and immune infiltrates in patients treated with TACE or SIRT could be related to several factors. First, the mechanisms leading to cell death are different in these two approaches. TACE uses larger microspheres (embolized at the arteriolar level) and produces ischemia-induced cell death, whereas SIRT uses smaller radioactive microspheres (embolized at the capillary level), and produces radiation-induced immunogenic cell death. Second, the kinetics of these two treatments may be different and it is possible that 15 weeks between intra-arterial treatment and surgery is not long enough for optimal visualization of significant tumor necrosis after SIRT [44, 45].

In the present study, confirming previous observations [26, 46–48], neither SIRT nor TACE were able to improve outcomes after PH as compared with patients receiving no preoperative treatment. Moreover, irrespective of the preoperative treatment, we did not observe any impact of the extent of TILs or of the intra-tumoral expression of GZB on recurrence rates and OS. While our study was underpowered to address this question, this may indicate that, under these conditions, locally-attracted immune cells may have not acquired full functionality, remaining unable to generate a robust anti-tumor response in the remnant liver. In fact, in HCC and in other tumors, the existence of non-redundant mechanisms of tumor resistance to radiation has been suggested by several observations, such as the variability of the responses and the extreme rarity of the abscopal effect [49, 50]. Interestingly, therapeutic combinations, such as radiotherapy and immunotherapy or dual checkpoint blockade may, at least partially, overcome these resistance mechanisms [51].

## Conclusion

These results should be interpreted cautiously due to the limited number of patients and the retrospective nature of the study. Furthermore, as TILs in SIRT-treated patients have not been functionally investigated, and as no immunotherapy was given in these patients, the real immunogenic effect and potential synergy with checkpoint inhibitors or other immunomodulators remain to be verified. Yet, together with recent observations [38, 46], our results suggest that the combination of SIRT with immunotherapy, such as checkpoint blockade or dual checkpoint blockade, may represent an attractive therapeutic strategy for treatment of HCC, either in a preoperative setting, before PH or RF, or for palliation in patients not amenable to curative-intent treatment. The absence of immunogenic effects of TACE observed here suggests that SIRT should be tested preferentially in investigational protocols combining intra-arterial treatments with immunotherapy. This approach, and its further refinements, including the definition of the optimal dose of irradiation, the type of immunotherapy and the optimal schedule for sequential interventions, requires further evaluation in prospective studies.

## Data Availability

The datasets generated and analyzed during the current study are available from the authors on reasonable request.

## Abbreviations

^90^Y: ^90^Yttrium
AFP: Alpha-fetoprotein
GZB: Granzyme B
HBV: Hepatitis B virus
HCC: Hepatocellular carcinoma
HCV: Hepatitis C virus
IHC: Immunohistochemistry
MELD: Model for end-stage liver disease
OS: Overall survival
PFS: Progression-free survival
PH: Partial hepatectomy
RF: Radiofrequency
SIRT: Selective internal radiation therapy
TACE: Transarterial chemoembolization
TIL: Tumor-infiltrating lymphocytes
